# Investigation of the Relationship Between Clinical Features and the Sonographic Appearance of the Ovaries with the Delta Neutrophil Index in Polycystic Ovary Syndrome

**DOI:** 10.5935/1518-0557.20250014

**Published:** 2025

**Authors:** Uğurcan Zorlu, Batuhan Turgay, Sezer Nil Yılmazer-Zorlu, Mohammad İbrahim Halilzade, İnci Halilzade, Hakan Raşit Yalçın

**Affiliations:** 1 University of Health Sciences Ankara City Hospital, Gynecology and Obstetrics Department, Ankara, Turkey; 2 Ankara University Faculty of Medicine, Gynecology and Obstetrics Department, Ankara, Turkey; 3 Ankara Mamak State Hospital, Radiology Department, Ankara, Turkey

**Keywords:** PCOS, infertility, ovarian volume, delta neutrophil index, hirsutism

## Abstract

**Objective:**

Polycystic Ovary Syndrome (PCOS) is a common endocrine disorder in women of reproductive age, characterized by metabolic, reproductive, and inflammatory disturbances. This study aimed to investigate the relationship between clinical features, the sonographic appearance of the ovaries, and the Delta Neutrophil Index (DNI) in women with PCOS.

**Methods:**

This retrospective study included 60 women diagnosed with PCOS and 60 healthy controls. Clinical, laboratory, and ultrasonographic data were collected and analyzed. The Delta Neutrophil Index (DNI), neutrophil/lymphocyte ratio (NLR), platelet/lymphocyte ratio (PLR), and ultrasonographic parameters, including ovarian stroma/total area ratio, and ovarian volume, were compared between the groups.

**Results:**

The study found that DNI, NLR, and PLR were significantly higher in the PCOS group compared to the control group, indicating a chronic low-grade inflammatory state. Significant differences were also observed in ovarian stroma/total area ratio, and ovarian volume between the groups. Additionally, a statistically significant relationship between acne and DNI was found in the PCOS group, while other symptoms such as infertility, hirsutism, and hair loss did not show a significant correlation with DNI.

**Conclusions:**

This study demonstrates a clear relationship between elevated inflammatory markers and ovarian morphology in PCOS. The findings suggest that inflammation plays a significant role in the pathophysiology of PCOS, underscoring the importance of incorporating anti-inflammatory treatment strategies in the clinical management of the disease.

## INTRODUCTION

Polycystic Ovary Syndrome (PCOS) is one of the most common endocrine disorders in women of reproductive age, characterized by anovulation, hyperandrogenism, and polycystic ovary morphology (Amsterdam ESHRE/ASRM-Sponsored 3rd PCOS Consensus Workshop Group, 2012). This syndrome adversely affects not only reproductive health but also metabolic and psychological well-being (Azziz *et al*., 2004). Women with PCOS commonly experience metabolic complications such as insulin resistance, dyslipidemia, obesity, and increased cardiovascular risk (Teede *et al*., 2018). Additionally, clinical symptoms such as infertility, hirsutism, acne, hair loss, and menstrual irregularities are frequently observed in these patients (Goodarzi *et al*., 2011).

Although the pathogenesis of PCOS is not yet fully understood, it is believed that the syndrome is associated with chronic low-grade inflammation (Rotterdam ESHRE/ASRM-Sponsored PCOS Consensus Workshop Group, 2004). In recent years, there has been an increase in studies investigating the use of hematological markers to evaluate this inflammatory response (Kelly *et al*., 2001). One such parameter is the delta neutrophil index (DNI), which measures the difference between neutrophil precursors and mature neutrophils and is considered a biomarker reflecting systemic inflammation (Kayacık Günday & Yılmazer, 2024). The role of DNI as an indicator of increased inflammatory response in women with PCOS, and its relationship with the clinical characteristics of the disease, is still under investigation (Barry *et al*., 2014; Azziz *et al*., 2006).

Furthermore, ultrasonographic parameters such as ovarian artery Doppler, ovarian stroma/total area ratio, and ovarian volume in PCOS patients provide significant data to better understand the clinical features of this syndrome (Michele *et al*., 2025). Ovarian artery Doppler measurements are particularly used to evaluate ovarian blood flow, and significant differences have been observed in these values in PCOS patients (Thessaloniki ESHRE/ASRM-Sponsored PCOS Consensus Workshop Group, 2008). The ovarian stroma/total area ratio and ovarian volume are also increased in women with PCOS, and statistically significant results have been obtained compared to the control group (Dewailly *et al*., 2014). These ultrasonographic parameters play an important role in the diagnosis of PCOS and in monitoring the progression of the disease.

The primary aim of this study is to compare delta neutrophil index values between women with PCOS and healthy individuals. Secondly, the relationship between delta neutrophil index and clinical symptoms such as infertility, hirsutism, acne, hair loss, and menstrual irregularities in women with PCOS will be examined. In addition, the differences in ultrasonographic parameters such as ovarian artery Doppler, ovarian stroma/total area ratio, and ovarian volume between PCOS patients and the control group will also be evaluated. These findings are expected to provide valuable insights for the clinical management and development of treatment strategies for PCOS.

## MATERIALS AND METHODS

This retrospective study includes the analysis of the clinical, laboratory, and ultrasonographic findings of 60 women diagnosed with PCOS and 60 healthy individuals in the control group. The study was conducted with local ethics committee approval and in accordance with the Helsinki Declaration.

### Study Group Selection

The diagnosis of PCOS was based on the 2003 Rotterdam criteria (Rotterdam ESHRE/ASRM-Sponsored PCOS Consensus Workshop Group, 2004). The study group was selected from women diagnosed with PCOS in our hospital between 2020 and 2023. The control group was composed of healthy individuals with similar demographic characteristics such as age and body mass index (BMI). None of the individuals in the control group had any endocrine or metabolic diseases.

### Data Collection

The clinical data of the patients were retrospectively collected from the hospital database. Demographic data, BMI, menstrual regularity, hirsutism, acne, hair loss, and other symptoms were recorded. Laboratory data included delta neutrophil index (DNI), complete blood count (CBC) parameters, and ultrasonographic findings (antral folicle count (AFC), ovarian stroma/total area ratio, and ovarian volume). The delta neutrophil index was calculated from peripheral blood samples using the formula (% band neutrophil + % segmented neutrophil) / % lymphocyte.

### Ultrasonographic Evaluation

Transvaginal ultrasound was used to measure AFC and ovarian volume. The ovarian volume, ovarian stroma/total area ratio were calculated from ultrasound images, and AFC was also recorded. All ultrasonographic measurements were performed by an experienced radiologist.

### Statistical Analysis

The data were analyzed using SPSS (Statistical Package for the Social Sciences) version 25.0. The normality of data distribution was assessed using the Shapiro-Wilk test. Normally distributed data were presented as mean±standard deviation (Mean±SD). Group comparisons for continuous variables were performed using the independent sample t-test or Mann-Whitney U test. A *p*-value of <0.05 was considered statistically significant.

## RESULTS

In this retrospective study, clinical, laboratory, and ultrasonographic data were compared between 60 women with Polycystic Ovary Syndrome (PCOS) and 60 healthy individuals in the control group.

### Demographic Data and Clinical Findings

The mean age of the patients in the PCOS group was 28.4±5.2 years, while the mean age of the individuals in the control group was 27.9±4.8 years, with no significant difference between the groups in terms of age and BMI (*p*>0.05). The most common clinical symptoms in the PCOS group were hirsutism (60%), acne (43%), hair loss (50%), and menstrual irregularities (73%).

### Delta Neutrophil Index and Complete Blood Count Parameters

The delta neutrophil index (DNI) was significantly higher in the PCOS group compared to the control group (*p*=0.04). The neutrophil/lymphocyte ratio (NLR) was also significantly higher in the PCOS group compared to the control group (*p*=0.043). Platelet count was 314.4±81.2 in the PCOS group and 290±67.85 in the control group, with this difference also being statistically significant (*p*=0.036). There was no significant difference between the groups in terms of lymphocyte count (*p*=0.059). However, the platelet/lymphocyte ratio (PLR) was 143.2±56.42 in the PCOS group and 117.2±54.42 in the control group, with this difference being statistically significant (*p*=0.023) (Table 1).

**Table 1:** Comparison of hemogram and delta neutrophil index values between the PCOS and control groups.

Parameter (n=60)	PCOS	Control (n=60)	p-value
Delta neutrophil index	0.35±0.066	0.31±0.081	0.040
Neutrophil/lymphocyte ratio	2.54±1.42	2.1±0.84	0.043
Platelet	314.4±81.2	290±67.85	0.036
Lymphocyte	2.2±0.81	2.5±0.89	0.059
Platelet/lymphocyte ratio	143.2±56.42	117.2±54.42	0.023

### Relationship Between Symptoms and Delta Neutrophil Index

When evaluated in terms of symptoms, a statistically significant relationship was found between the presence of acne and the delta neutrophil index in the PCOS group (*p*=0.001). Patients with acne had a higher delta neutrophil index. There was no significant relationship between delta neutrophil index and infertility, hirsutism, hair loss, or menstrual irregularities (Table 2).

**Table 2:** Relationship between symptoms and delta neutrophil index in the PCOS group.

Symptom	Delta Neutrophil Index		p-value
	(Present)	(Absent)	
Infertility	0.33±0.071	0.32±0.062	0.121
Acne	0.38±0.059	0.31±0.069	0.001
Hirsutism	0.33±0.067	0.33±0.006	0.144
Hair loss	0.32±0.058	0.34±0.065	0.091
Menstrual irregularities	0.34±0.057	0.32±0.065	0.072

### Ultrasonographic Findings

A total of 120 patients were included, with 60 in the PCOS group and 60 in the control group. The parameters evaluated include the mean ovarian stromal area relative to the ovarian area, mean ovarian volume, and mean antral follicle count. The results are summarized in Table 3.

**Table 3:** Ultrasonographic findings between the PCOS and control groups.

Parameter	PCOS (n=60)	Control (n=60)	p-value
Mean ovarian stromal area/ovarian area	0.44±0.21	0.32±0.11	<0.001
Mean ovarian volume (cm^3^)	12.2±2.1	8.8±0.8	<0.001
Mean Antral Folicule Count	14.4±6.1	7.1±5.2	<0.001

### Mean Ovarian Stromal Area/Ovarian Area

The mean ovarian stromal area to ovarian area ratio was significantly higher in the PCOS group (0.44±0.21) compared to the control group (0.32±0.11), with a *p*-value of <0.001. This indicates a significant increase in the stromal area in patients with PCOS, suggesting that the ovarian stroma is more prominent relative to the total ovarian area in this population.

### Mean Ovarian Volume

The mean ovarian volume was also significantly greater in the PCOS group (12.2±2.1cm^3^) compared to the control group (8.8±0.8 cm^3^), with a *p*-value of <0.001. This finding reflects the characteristic enlargement of the ovaries often observed in PCOS patients, which is typically attributed to the presence of multiple follicles and increased stromal tissue.

### Mean Antral Follicle Count

Additionally, the mean antral follicle count was significantly higher in the PCOS group (14.4±6.1) compared to the control group (7.1±5.2), with a *p*-value of <0.001. This result underscores the hyperandrogenic state and disrupted follicular development commonly associated with PCOS, where an increased number of antral follicles is noted.

### DISCUSSION

PCOS is a common endocrine disorder in women that affects multiple systems, including reproductive health and metabolic processes. In our study, we found that inflammatory parameters such as DNI, NLR, platelet count, and PLR were significantly higher in women with PCOS compared to the control group. These results support the presence of chronic low-grade inflammation in women with PCOS, which is consistent with findings from previous studies in the literature (Rudnicka *et al*., 2021).

### Delta Neutrophil Index and Inflammation

DNI is used as a significant marker of inflammation. In a study conducted on sepsis patients, a DNI cutoff value of 0.027 was found to have high sensitivity (89%) and specificity (86%) for distinguishing septic patients (Park *et al*., 2011). Furthermore, in patients with systemic inflammatory response syndrome (SIRS), a DNI cutoff value of 0.0275 was used to predict candidemia, demonstrating a sensitivity of 72.9% and a specificity of 78.6% (Park *et al*., 2020). In liver transplant recipients, a DNI value of 0.0205 on postoperative day 14 was identified as an important prognostic marker for predicting early mortality (Lee *et al*., 2023).

DNI has been investigated as a potential marker for inflammation in PCOS. A study by Gunday and Yılmazer found that the DNI was significantly higher in PCOS patients compared to controls, with a suggested cutoff value of 0.015 for distinguishing between the two groups. This cutoff demonstrated a sensitivity of 78% and a specificity of 35%, with an area under the curve (AUC) of 0.588 (Günday & Yılmazer, 2023).

In our study, the significantly higher DNI values in women with PCOS compared to the control group indicate the presence of chronic low-grade inflammation in these patients. Moran *et al*. (2015) similarly reported increased inflammatory responses in women with PCOS, linking this increase to metabolic complications such as insulin resistance and obesity. It is frequently emphasized in the literature that inflammation contributes to the clinical features of PCOS, including insulin resistance and hyperandrogenism (Legro *et al*., 2013).

NLR is another indicator of inflammatory response, and it was also found to be significantly higher in women with PCOS compared to the control group. This finding has been confirmed by studies such as that by Li *et al*. (2022). Elevated NLR levels suggest that inflammation is widespread in women with PCOS and may be related to the metabolic and cardiovascular complications of the disease (Diamanti-Kandarakis & Dunaif, 2012).

### Platelet Count and Platelet/Lymphocyte Ratio (PLR)

Our study also observed that platelet count and PLR were significantly higher in women with PCOS compared to the control group. This finding strengthens the role of inflammation in the pathogenesis of PCOS. Similar results were reported by Talmor & Dunphy (2015). Increased PLR values indicate that inflammatory processes are becoming more pronounced, and PCOS is associated with chronic inflammation (Rudnicka *et al*., 2021). Elevated PLR levels suggest that women with PCOS are at an increased risk for cardiovascular complications, which may lead to long-term health issues (Özay & Özay, 2021).

### Inflammation Associated with Symptoms

In this study, we evaluated the relationship between symptoms and delta neutrophil index, and found a significant correlation between the presence of acne and DNI in the PCOS group. Acne is an inflammatory skin condition, and inflammation may have significant effects on the skin in women with PCOS (Tanghetti, 2013). However, no significant relationship was found between DNI and infertility, hirsutism, hair loss, or menstrual irregularities. These results suggest that inflammation is more related to metabolic processes, and hormonal imbalances play a larger role in the emergence of these symptoms (Barber *et al*., 2006).

### Ultrasonographic Findings and Ovarian Morphology

The findings from this study highlight the distinct ultrasonographic characteristics of PCOS. Notably, the ovarian stromal area to ovarian area ratio and mean ovarian volume were significantly higher in the PCOS group compared to controls. These results are consistent with previous research indicating that increased stromal tissue and enlarged ovarian volume are characteristic features of PCOS (Buckett *et al*., 1999).

Additionally, the mean AFC was significantly elevated in the PCOS group. This aligns with the established understanding that women with PCOS often exhibit a higher number of antral follicles, which reflects the disrupted follicular development associated with the syndrome (Franks & Hardy, 2020). The increased AFC, combined with the heightened stromal area and ovarian volume, reinforces the notion that ultrasonographic parameters are valuable in the diagnosis and assessment of PCOS.

In summary, our study underscores the significance of ultrasound findings, particularly ovarian volume, stromal area, and AFC, in characterizing PCOS. These metrics not only assist in diagnosis but also enhance our understanding of the pathophysiology of this condition, potentially guiding future management strategies.

## CONCLUSION

This study demonstrates that inflammatory markers (DNI, NLR, PLR) are elevated in women with PCOS, and significant changes in ovarian morphology are observed. It is clear that inflammation is an important factor that should be considered in the clinical management of PCOS. Given that PCOS affects not only reproductive health but also metabolic processes and cardiovascular health, it is of great importance to develop treatment strategies that target inflammation. Further prospective studies are needed to better understand the effects of inflammation on PCOS. These findings suggest that targeting inflammation in treatment strategies may be crucial in preventing long-term complications associated with PCOS.
